# Study on the Memory Effect in Aerosil-Filled Nematic Liquid Crystal Doped with Magnetic Nanoparticles

**DOI:** 10.3390/nano13232987

**Published:** 2023-11-21

**Authors:** Peter Bury, Marek Veveričík, František Černobila, Natália Tomašovičová, Veronika Lacková, Katarína Zakutanská, Milan Timko, Peter Kopčanský

**Affiliations:** 1 Department of Physics, Žilina University, Univerzitná 1, 010 26 Žilina, Slovakia; 2 Institute of Experimental Physics, Slovak Academy of Sciences, Watsonova 47, 040 01 Košice, Slovakiagdovinova@saske.sk (V.L.);

**Keywords:** liquid crystals, aerosil nanoparticles, magnetic nanoparticles, memory effect, light transmission, SAW attenuation responses

## Abstract

A study on 5CB liquid crystal composites with SiO_2_ nanoparticles and an additional commixture with Fe_3_O_4_ nanoparticles using light transmission and SAW measurements is presented. The prepared liquid crystal composites exhibited an interesting memory effect characterized by the hysteresis of both light transmission and SAW attenuation responses investigated in the nematic phase. While in the case of SiO_2_ nanoparticles as dopants, the liquid crystal composite showed an improvement in the memory effect, the addition of Fe_3_O_4_ magnetic nanoparticles resulted in the memory effect decreasing. Additional studies showed a significant shift in both the threshold voltage and nematic–isotropic transition temperature. Measurements in the magnetic field confirmed the increasing memory effect according to that of pure 5CB. The properties of these composites could lead to a potential application for the fabrication of memory devices suitable for information storage.

## 1. Introduction

The existence of the memory effect in soft materials has a significant role in a wide range of applications in the electronic industry. Physical properties connected with electronic data storage have drawn a great deal of research interest in both fundamental and applied physics and remarkable physical phenomena were exhibited [1,2,3,4]. Liquid crystal (LC) utilization for the storage of electronic data has further inspired the interest of research groups [5,6,7,8,9], and nanoparticles of an inorganic nature dispersed in LC matrices due to the interactions between LCs and nanoparticles provide great potential for this application [10,11,12,13,14]. For instance, non-volatile memory effects have been studied using low-frequency dielectric spectroscopy in the nematic phase of ferroelectric LCs doped with polymer-capped gold nanoparticles [15]. The presence of ferroelectric nanoparticles like barium titanate (BaTiO_3_) gives rise to comparable results in the electro-optical properties of LC composites and dielectric measurements [16,17,18,19,20]. Furthermore, it has been found that nanoparticles can significantly improve some LC properties, as the presence of carbon nanotubes properly influences LC electro-optic responses [21], quantum dots modify the chiral pitch in cholesteric LCs [22], and ferroelectric nanoparticles can significantly enhance the orientational order and, through that, the electro-optic response, including a reduction in the threshold voltage [23,24,25,26].

More recently, dielectric experiments using functionalized barium titanate as ferroelectric nanoparticles dispersed in 5CB [20,27] detected a giant soft memory effect. However, these experiments were conducted in the isotropic phase, which rather limited the potential application of such composites because of the required relatively high electric fields. This is the reason that additional modifications of LCs and nanoparticles, as well as their impact on LC properties, represent a continual active area in current research. Silica (also known as aerosil) nanoparticles dispersed into nematic LCs draw remarkable attention because the material was doped to nematic LCs as the first type of dielectric nonmagnetic and inorganic nanoparticles [28]. Aerosil’s interesting properties also allow the formation of prospective applications in electronic devices based on electro-optic and memory effects [28,29,30,31]. The interesting aspect of aerosil–LC composites is the role of other kinds of nanoparticles in tuning their memory properties.

The present contribution continues the preliminary investigation of the memory effect within the scope of dielectric properties in 5CB liquid crystals doped with SiO_2_ nanoparticles and their mixture with Fe_3_O_4_ nanoparticles investigated using capacitance–voltage measurements [32]. The prepared LC (5CB) compounds are investigated in the nematic phase with the intention of hysteresis behavior after only small voltages and weak magnetic fields are applied. Results reported here were obtained for the first time using both light transmission measurements and surface acoustic wave (SAW) attenuation response measurements. The connection between these techniques has provided interesting results in the last decade [33,34,35].

## 2. Experimental Methods

The LC composites were based on 5CB nematic liquid crystals (with a nematic–isotropic transition temperature of 35 °C) and silica nanoparticles, Aerosil R812 (~7 nm) nanoparticles, obtained from Evonik Industries (Essen, Germany). Spherical Fe_3_O_4_ nanoparticles with an average dimension of ~10 nm were obtained from Ocean Nanotech (Springdale, AR, USA). The SiO_2_ nanoparticles (0.04 g) were dispersed in 5CB (3.96 g) to obtain 1.0 wt.% of particles in solution. A particular amount of Fe_3_O_4_ nanoparticles embedded in chloroform was added to a sample of the 5CB composite containing SiO_2_ at 1 wt.% to obtain the specific volume fraction of Fe_3_O_4_ nanoparticles. This sample was sonicated in the isotropic phase for 4 h, allowing the chloroform to evaporate. The composite with a concentration of 1.0 wt.% was then, in this way, prepared, and the sample with a concentration of 0.5 wt.% was worked off by mixing in an additional amount of 5CB. As composites with a low concentration of SiO_2_, similar to undoped 5CB, they showed only small dielectric hysteresis under the applied electric field, and larger hysteresis was observed only with the higher SiO_2_ wt.% concentration [32], which could stabilize more LC molecules in the vertical alignment. The latter composites were prepared for the following investigation. Concerning the Fe_3_O_4_ nanoparticle concentration, the reason is similar because it was found that while a low volume fraction of this impurity (10^−4^) increases the size of hysteresis, its further increase reduces the memory effect in 5CB.

The role of SiO_2_ nanoparticles as well as Fe_3_O_4_ nanoparticles in LC structural changes including the memory effect and sequent development of optical properties were investigated using light transmission measurements. The LC cells used for experimental investigation were similar to those used in capacitance measurements (*D* = 50 μm) [32]. Briefly, commercial cells from MWAT (Warsaw, Poland) were coated with ITO transparent conductive layers. To ensure parallel alignment, the layers were rubbed in a direction parallel to the electrodes. A capillary method was used to fill the cells but in the LC isotropic phase. A green laser beam (532 nm, 5 mW) illuminated the cell’s glass under normal incidence. An optical polarizer ensured the linearly polarized incident light beam in the initial position for all investigated composites. The LC cell position guarantees maximal (in the arrangement of parallel polarizers) or minimal (in the arrangement of crossed polarizers) transmittance. The intensity of transmitted light was registered with a photodetector and subsequently recorded by a computer as the light transmission dependence on electric or magnetic fields. The complete experimental arrangement configuration was already described in [33,34]. The light transmission in the case of parallel polarizers was then expressed as *I*/*I*_0_, and in the case of crossed polarizers, as (*I* − *I*_0_)/*I*_0_, where *I* was the measured light intensity and *I*_0_ was the maximal intensity of incident light passing through the LC.

The investigation of the attenuation response of a surface acoustic wave (SAW) was used as another technique to verify the influence of SiO_2_ and/or Fe_3_O_4_ nanoparticles on the memory effect after the electric field was applied. The cells of the investigated LC composites with a thickness of *D* ≈ 100 μm were prepared on the center of the LiNbO_3_ piezoelectric line. The line was supplied by two interdigital transducers. The experimental arrangement and measurement procedure were also already described in [33,35]. Concisely, SAW pulses with a frequency of 20 MHz and a pulse width of ~1 μs were generated with one of the interdigital transducers using hf pulses. Another transducer was used to receive the SAW signal, using both a Pulse Generator and a Receiver of MATEC 7700. A MATEC Attenuation Recorder 2470 was used to record the SAW attenuation response. In the LC cells, for SAW measurement, the initial intrinsic arrangement of LC molecules and by that director ***n*** were supposed to have a predominant alignment in the plane of the LC cell. Then, electric and magnetic fields were applied across the LC cell. The amplitude of the SAW after reaching the LC layer was attenuated because the SAW radiates into LC in a longitudinal wave, giving rise to propagation losses [35,36]. Due to the absorption of the longitudinal wave by the LC structure, the SAW attenuation *α* subsequently responds to any structural changes in an LC composite initiated by external conditions including electric and magnetic fields. The instabilities for the measurement of Δ*α* were better than ±0.02 dB. The temperature of the sample holder, the accuracy of which was better than ±0.2 °C, could be stabilized in the range of 5–80 °C.

## 3. Results and Discussion

### 3.1. Light Transmission Investigation

**Figure 1:**
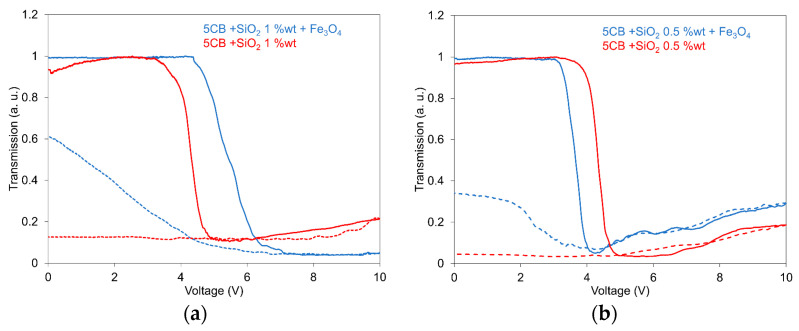
Light transmission dependence on the electric field for the 5CB liquid crystal doped with SiO_2_ nanoparticles at concentrations of 1.0 wt.% (**a**) and 0.5 wt.% (**b**) including their mixture with Fe_3_O_4_ nanoparticles with a volume fraction of 10^−4^ measured in increasing (full lines) and decreasing regimes (dotted lines). The measurements were conducted at 25 °C.

The light transmission was investigated, using the arrangement outlined in the previous paragraph, in the set of 5CB composites doped with SiO_2_ nanoparticles of concentrations 1.0 wt.% and 0.5 wt.%, respectively. The composites contained, in addition to their mixture with Fe_3_O_4_, nanoparticles with a volume fraction of 10^−4^. The light transmission measurements are presented for the arrangement with parallel polarizers when the light transmission was expressed as *I*/*I*_0_, where *I*_0_ is the maximal intensity of incident light passing through the LC and *I* is the intensity under applied field. Figure 1a,b illustrates the dependences of light transmission on the electric field for 5CB composites with SiO_2_ nanoparticles at concentrations of 1.0 wt.% (Figure 1a) and 0.5 wt.% (Figure 1b) that are compared with composites containing their mixture with Fe_3_O_4_ nanoparticles with a volume fraction of 10^−4^, measured in both increasing and decreasing regimes. All presented characteristics show similar development for an increasing electric field, characterized by an almost constant light transmission lasting until 3.0–4.5 V, depending on the nanoparticle concentration, followed by a rapidly decreasing characteristic for the threshold field. After reaching their minimum, either a constant or only a small increase in the light transmission was registered for voltage increasing up to its maximal measured value (10 V). However, the light transmission development for the decreasing voltage, when a large memory effect occurs, was for composites containing only SiO_2_ aerosil, which are evidently different from composites with added Fe_3_O_4_ nanoparticles. While SiO_2_ nanoparticles at both concentrations, i.e., 1.0 wt.% and 0.5 wt.%, practically cause the freezing of structural changes induced by the external voltage, creating an almost non-transparent layer, their mixing with Fe_3_O_4_ nanoparticles with a volume fraction of 10^−4^ disrupts this property; however, the large memory effect permanently remains. The role of Fe_3_O_4_ nanoparticles in light transmission measurements appears to be the same as in previous dielectric measurements [33]: the memory effect was repressed.

**Figure 2:**
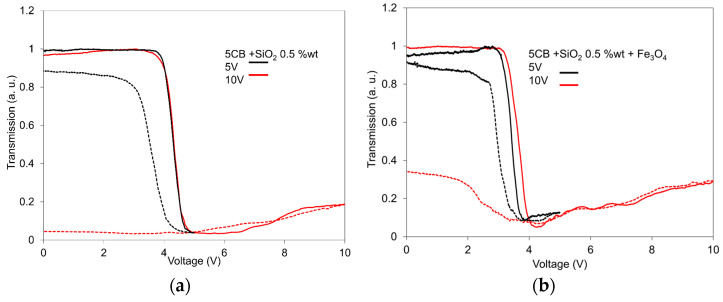
Comparisons of light transmission dependences on the electric field for the 5CB liquid crystal doped with SiO_2_ nanoparticles at a concentration of 0.5 wt.% (**a**) and its mixture with Fe_3_O_4_ nanoparticles with a volume fraction of 10^−4^ (**b**) measured for two different voltage ranges: 0–5 V and 0–10 V, in increasing (full lines) and decreasing regimes (dotted lines). The measurements were conducted at 25 °C.

The comparisons of light transmission dependencies on the electric field for the 5CB composite with SiO_2_ nanoparticles at concentrations of 0.5 wt.% and the same composite containing extra Fe_3_O_4_ nanoparticles, as representative samples measured at two different voltage ranges, i.e., 0–5 V and 0–10 V, are presented in Figure 2a,b. The essential feature of these comparisons is that the measurements were realized only to the maximal value of 5 V, even passing through the threshold field, showing only a very low memory effect in contrast with the situation when the voltage increases up to 10 V. This fact coincides with most of the SAW measurements results, in which irreversible structural changes are registered after reaching some value of the electric field. The process of structural changes continues even at decreasing fields. This aspect of LC composite behavior could be explained with the expectation that for the first run of driving, realized only to 5 V, only some fraction of the random network creates the organized structure because the driving voltage is not high enough. The random network continues in the creation of the organized network during the next increase in the driving voltage up to 10 V, and the memory effect corresponds to the new order.

The memory effect in investigated nematic LC composites can be explained by the process of the formation of some kinds of network structure in the LC matrix [37,38]. The responsibility for the network creation, including holding the network together, is attributed to hydrogen bonds among the aerosil surface as well as between the aerosil and LC molecules [39,40]. After the application of the electric field to the still non-ordered composite, the intrinsic arrangement follows the alignment of the created nematic domains. The resulting network then has an anisotropic structure with an orientation in the direction of the electric field and keeps the director ***n*** parallel to the vector ***E***. Consequently, the SiO_2_ nanoparticles form a network in the LC matrix, that changes from a random to partially organized one under the influence of an electric field, and the alignment is stable after the driving field elimination. So, the stabilization of the LC domain remains even when the electric field is removed. Furthermore, the magnitude of the memory effect depends on the number of surface groups available to form bonds with liquid crystal molecules, and with that, it increases with increasing aerosil concentration [40]. The anchoring strength of LC molecules at the aerosil surface, characterized by a homeotropic nature, is a fundamental condition for the memory effect to take place [39,41]. Concerning Fe_3_O_4_ nanoparticles that have a spherical shape, they are characterized by soft and parallel anchoring to LC molecules [35,42].

However, the observed memory effect depends on the process of composite heating and cooling back to a measuring temperature. The higher rate of heating and rapid cooling result in more pronounced memory. The effect can be explained by the breaking and subsequent reforming of the network [43]. The memory effect subjected to an aerosil network is different from that registered in LC composites doped with quantum dots, where the memory is based on capture and/or emission barrier for quantum dots. However, some similarities can be found [44].

When Fe_3_O_4_ nanoparticles are presented in 5CB composites with SiO_2_ nanoparticles, the memory effect is reduced, provided that the reduction of *H*-bond is responsible for this effect. It is supposed that magnetic nanoparticles act as impurities in the investigated composites, causing partial decay of the organized network, especially the *H*-bonds created among both aerosil particles and between particles and LC molecules. Certainly, the presence of hydrogen bonds in the system strongly affects the efficiency of the memory effect, and the size of the memory effect is influenced by the number of OH groups at the surface that are available to form *H*-bonds with the LC molecules [39,40].

It should be noted that in the increasing voltage regime, a laser beam scattering was registered in the light transmission measurements, probably due to the domain structure of the LC matrix created by aerosil agglomerates [40], at which the size of domains and the inter-particle distances are considerably influenced by the overall aerosil concentration. However, LC molecules under the applied electric field change their orientation toward the field direction, which may result in aerosil clusters. This reorientation process can then be registered through light transmission changes.

**Figure 3:**
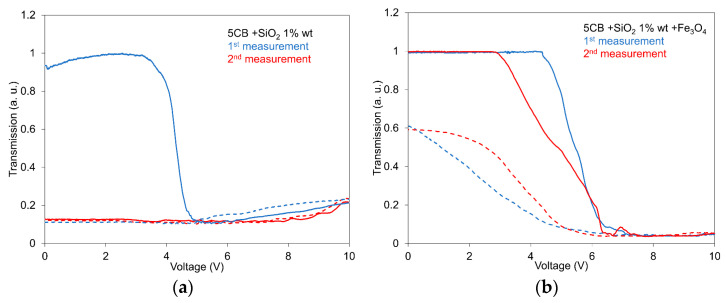
Light transmission dependences on the electric field for the 5CB composition with SiO_2_ nanoparticles at a concentration of 1.0 wt.% (**a**) including their mixture with Fe_3_O_4_ nanoparticles with a volume fraction of 10^−4^ (**b**) measured in the electric field to compare their development measured one after another in interval 1.0 h in increasing (full lines) and decreasing regimes (dotted lines). The measurements were conducted at 25 °C.

Figure 3a,b presents the light transmission dependencies of the 5CB composites with SiO_2_ nanoparticles at a concentration of 1.0 wt.% (a) and their mixture with Fe_3_O_4_ nanoparticles with a volume fraction of 10^−4^ (b) on the electric field measured one after another in an interval of 1.0 h. The development of the light transmission is here illustrated for two measurements indicating the behavior of memory storage in the investigated composites after some time interval. While the memory storage in the case of 5CB doped only with SiO_2_ nanoparticles (Figure 3a) remains practically conserved after 1 h, in the case of its mixture with Fe_3_O_4_ nanoparticles (Figure 3b), the characteristics indicate a return almost to the initial state, including the resulting memory storage. However, the individual light transmission dependencies were slightly different, which means the second measurement compared with the first one.

The presented dependencies again supported results concerning the effect of Fe_3_O_4_ nanoparticles on the storage effect in the investigated composites. Namely, Fe_3_O_4_ nanoparticles in a mixture of 5CB with SiO_2_ nanoparticles reduce the memory effect due to the partial disruption of the network structure in the LC matrix, originally created by aerosil nanoparticles and LC molecules. Particularly, it is due to the reduction of the *H*-bond responsible for the formation of the network [32].

**Figure 4:**
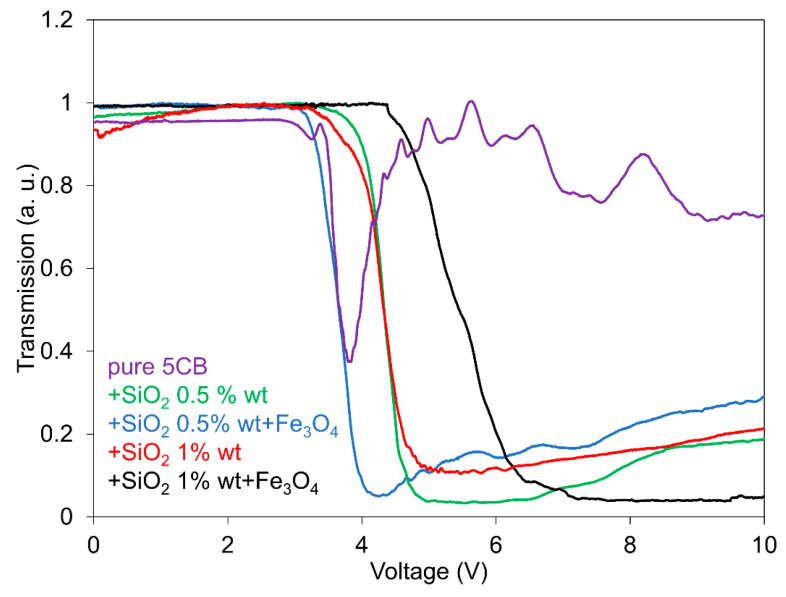
Summarization of light transmission dependences on the electric field for all the investigated 5CB composites, including pure 5CB, measured in an increasing regime. The measurements were conducted at 25 °C.

A summary of the light transmission dependencies on the electric field registered for all the investigated 5CB composites with SiO_2_ nanoparticles (1.0 wt.% and 0.5 wt.%) and their mixtures with Fe_3_O_4_ nanoparticles (10^−4^), including pure 5CB, measured only in increasing regimes, is shown in Figure 4. In the presented summary, the influence of added nanoparticles on the LC threshold voltage is evident. While the silica aerosil causes a shift in the threshold voltage for both concentrations toward higher fields in a similar manner, compared with pure 5CB, magnetic nanoparticles markedly shift the threshold voltage more, but with a different trend. The anomalous shift in the threshold voltage for different SiO_2_ concentrations in this case can be influenced by the different structures of SiO_2_ domains and their environment created by both LC molecules and Fe_3_O_4_ nanoparticles. While the SiO_2_ nanoparticles form a network in the LC matrix that is changed to the partially organized system under an electric voltage, dispersed magnetic Fe_3_O_4_ nanoparticles act as impurities, causing a disruption of the organized network in this system, mainly the H-bond network formed between the SiO_2_ nanoparticles and LC molecules. In the case of a lower concentration of SiO_2_ nanoparticles (0.5 wt.%), the Fe_3_O_4_ nanoparticles reduce both the memory effect and the threshold voltage, analogous to spherical nanoparticles [45]. For the higher concentration (1.0 wt.%), the memory effect is reduced similarly to the previous one; however, the threshold field increases further. It seems to be understandable that in this case, the Fe_3_O_4_ nanoparticles have different influences on the threshold voltage shift. Nevertheless, similar behavior was registered in 6CB liquid crystals dispersed with superionic nanoparticles [33]. Usually, an increase in the threshold voltage could be caused by an increase in the elastic constant of doped LC. The reason for such behavior at higher SiO_2_ concentrations can be then caused by different structures of SiO_2_ domains, including their vicinity formed by Fe_3_O_4_ nanoparticles.

**Figure 5:**
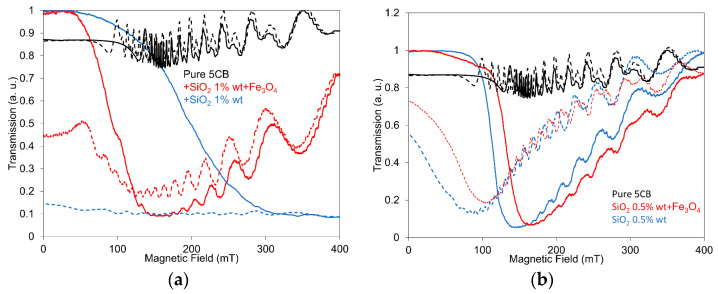
Light transmission dependencies on the magnetic field for the 5CB liquid crystal doped with SiO_2_ nanoparticles at a concentration of 1.0 wt.% (**a**) and 0.5 wt.% (**b**) including their mixture with Fe_3_O_4_ nanoparticles with a volume fraction of 10^−4^ measured in increasing (full lines) and decreasing regimes (dotted lines). The measurements were conducted at 25 °C.

The development of light transmission dependence on the magnetic field for the 5CB composites with SiO_2_ nanoparticles at both concentrations (1.0 wt.% and 0.5 wt.%), including their mixture with Fe_3_O_4_ nanoparticles with a volume fraction of 10^−4^ as well as pure LC, are presented in Figure 5a,b. Similar to the case of the applied electric field, after the magnetic field was applied to the investigated 5CB composites, strong memory effects could be observed. However, it was considerably influenced by the presence of both SiO_2_ and Fe_3_O_4_ nanoparticles. Measurements in the magnetic field again confirmed the fact that while the SiO_2_ aerosil markedly gives rise to the memory effect, the presence of Fe_3_O_4_ nanoparticles represses this effect. In addition, Fe_3_O_4_ nanoparticles contribute to the highlighting of 5CB magnetic properties including an oscillatory behavior. The lower SiO_2_ concentration (0.5 wt.%) and Fe_3_O_4_ nanoparticles enable the demonstration of magnetic properties of both 5CB and magnetic nanoparticles. It is interesting that the addition of only SiO_2_ nanoparticles to pure LC results in an increasing influence of the magnetic field on structural changes compared with pure 5CB. Concerning the light transmission oscillations at an external field higher than the threshold field, when the LC is subjected simultaneously to a laser beam, they are induced by a reorientation of the molecular nematic director ***n***. The explanation is then attributed to the directional redistribution of LC molecules around the equilibrium position of the director when it changes orientation from the initial planar alignment to the perpendicular one [45,46]. The same situation occurs when ***B*** is turned back to zero and LC molecules return to their initial orientation. Regarding the threshold voltage, it increased compared with pure LC in both SiO_2_ concentrations (1.0 wt.% and 0.5 wt.%); however, the addition of Fe_3_O_4_ nanoparticles shifted the threshold voltage to a higher voltage in the case of 1.0 wt.% and to lower voltage for the 0.5 wt.% concentration, similarly to the case when the electric field was applied. When similar samples were exposed to magnetic field *B* = 1 T for 5 min in the nematic phase [32], the memory effect was deleted. Such behavior does not correspond to our experimental results; however, we used a magnetic field only up to 400 mT.

### 3.2. SAW Investigation

The initial intrinsic arrangement of LC molecules, in the case of the SAW attenuation measurement, is supposed to have a predominate alignment in the plane of the LC cell, and the director ***n*** is thus parallel to the cell surface. The applied electric and/or magnetic fields were orientated perpendicularly to the cell plane. Due to the coupling between the dipole and/or magnetic moments of nanoparticles and LC molecules, the external fields turn the LC molecules also to the perpendicular direction. As the LC cell is prepared on the SAW path, the SAW amplitude after reaching the LC layer is attenuated due to the radiation of a longitudinal wave into the LC by the SAW. This gives rise to the SAW attenuation losses [35]. Considering that absorption of the longitudinal wave generated into the LC is influenced by the LC properties, including its structure, the measured SAW attenuation α can subsequently respond to any changes in the LC structure initiated by external fields or temperature. When our previous experimental results were compared with the theoretical consideration based on the hydrodynamic approximation [35], a unique indication followed, namely, that the bulk viscosity coefficients dominate in the SAW attenuation registered in this way, similar to the case of the longitudinal acoustic wave.

**Figure 6:**
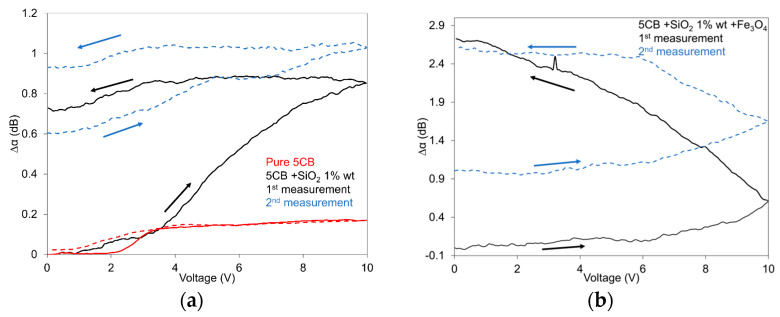
SAW attenuation response of the 5CB liquid crystal doped with SiO_2_ nanoparticles at a concentration of 1.0 wt.% (**a**) and their mixture with Fe_3_O_4_ nanoparticles with a volume fraction of 10^−4^ (**b**) on the electric field, including pure 5CB (**a**). The dotted lines represent the second measurement after 20 min (**a**) and 1.5 h (**b**), respectively. The measurements were conducted at 25 °C. Arrows denote increasing and/or decreasing regimes.

The effects of the external electric field on the SAW attenuation response in the 5CB composites with SiO_2_ nanoparticles at a concentration of 1.0 wt.%, including their mixture with Fe_3_O_4_ nanoparticles with a volume fraction of 10^−4^, in increasing and decreasing regimes, reflecting structural changes, are shown in Figure 6a,b. The development of the SAW attenuation response is illustrated here for two measurements one after another, pointing to the progress in the memory effect in the investigated 5CB composites. The results correspond to previous results obtained using capacitance measurements [32]. It is interesting that when the second measurement was performed in a relatively short time after the first one (20 min), the application of an electric field induced additional structural changes, continuing in the previous state formed after the first cycle (see Figure 6a). Nevertheless, when the second measurement was performed longer time after the first measurement (1 h), when the part of the structural changes caused by the first cycle was decayed, the repeated application of the electric field again induced the structural changes provided after the first cycle (see Figure 6b). The presented dependencies also show the particular role of both SiO_2_ aerosil and magnetic nanoparticles on the total structural changes characterizing the memory effect. While obvious dependences in nematic LCs [33,47] consist of three main regions: the nearly constant attenuation at lower voltages, the rapid increase after passing the threshold voltage, and the gradual approach to the saturation (see also pure 5CB development in Figure 6a), in the presented dependencies, only one development (samples with SiO_2_—first run) could correspond to the expected development. A characteristic feature of the presented dependencies is the continued increase in the SAW attenuation response in the decreasing regime in composites with Fe_3_O_4_ nanoparticles. The same situation was registered for composites with 0.5 wt.% of SiO_2_, but it was characterized by a weaker memory effect. The repeated measurements taken after some time (~1 h) confirmed some residual SAW attenuation, and by that, persisting structural changes. Concerning the threshold voltage, it could not be registered in this case due to the specific SAW attenuation developments that should correspond to structural changes, including the creation of domain structures by SiO_2_ nanoparticles that form a network in the LC matrix.

**Figure 7:**
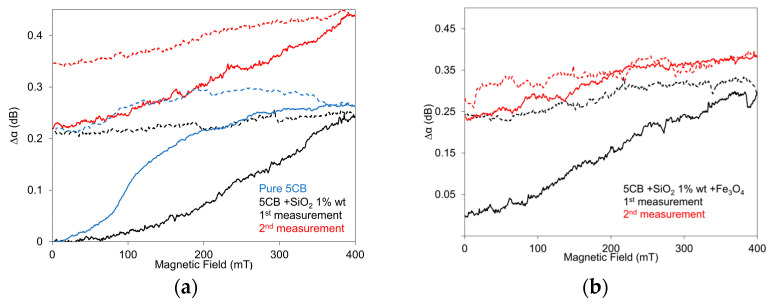
SAW attenuation response of the 5CB liquid crystal doped with SiO_2_ nanoparticles with a concentration of 1.0 wt.% (**a**) and their mixture with Fe_3_O_4_ nanoparticles with volume fraction 10^−4^ (**b**) on the magnetic field, including pure 5CB (**a**). The measurements were conducted at 25 °C. Red lines represent the second measurement after 1.5 h (**a**) and 20 min (**b**), respectively, measured in increasing (full lines) and decreasing regimes (dotted lines).

The behavior of the 5CB liquid crystal composites doped with SiO_2_ nanoparticles at a concentration of 1.0 wt.% and their mixture with Fe_3_O_4_ nanoparticles with a volume fraction of 10^−4^ in the magnetic field is illustrated in Figure 7a,b The total structural changes in these composites are for the first run similar to those observed in the case of pure 5CB, but the threshold field, similar to the case of the electric field, can be hardly distinguished. It is also evident that the presence of SiO_2_ aerosil dispersed into nematic 5CB suppressed some magnetic properties of pure LC. Concerning the memory effect, the measured dependences confirmed that structural changes are frozen for a certain time (see repeated measurement after 20 min, Figure 7b) but after a longer time (see repeated measurement after 1.5 h, Figure 7a), some defrosting of the LC structure probably occurs because under the following magnetic field, the network changes continue to form an organized structure. A similar development was observed for composites with aerosil at a concentration of 0.5 wt.%. The memory effect in pure LC can be attributed to structural deformation.

**Figure 8:**
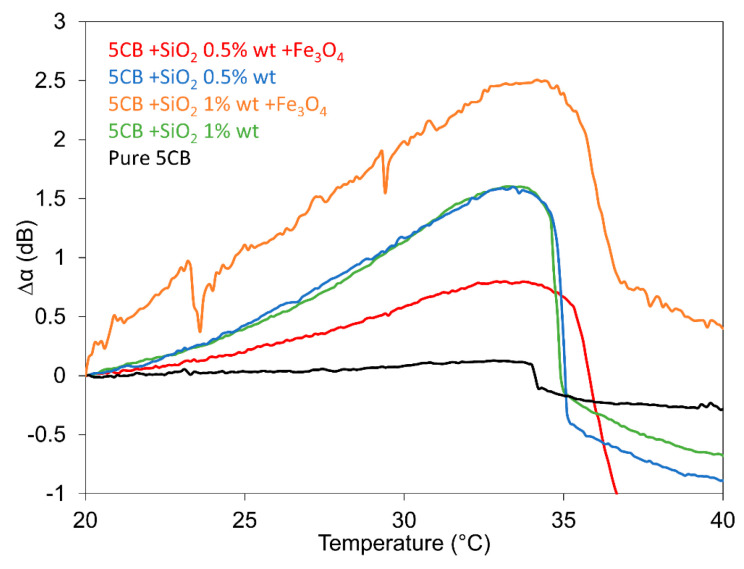
Temperature dependences of the SAW attenuation response for all investigated 5CB composites, doped with SiO_2_ nanoparticles at concentrations of 0.5 and 1.0 wt.% as well as their mixture with Fe_3_O_4_ nanoparticles with a volume fraction of 10^−4^, including pure 5CB.

The dependences of SAW attenuation on temperature for all the investigated 5CB composites consisting of SiO_2_ nanoparticles at concentrations of 0.5 and 1.0 wt.% as well as their mixture with Fe_3_O_4_ nanoparticles with a volume fraction of 10^−4^ including pure 5CB, focused on nematic–isotropic phase transitions, are illustrated in Figure 8. All the presented dependencies suggest very similar progress. This means slowly increasing attenuation from room temperatures until the temperature is close to ~32 °C after reaching the maximum value of SAW attenuation. Its subsequent rapid drop is registered, corresponding to the nematic–isotropic transition (*T_NI_*). The characteristic feature of temperature dependencies is the increase in the transition temperature *T_NI_* in composites caused by the addition of the silica aerosil. However, the presence of magnetic particles leads to an additional increase in transition temperature, despite the fact that spherical nanoparticles in the LC should reduce *T_NI_* [35]. However, the shift in the nematic–isotropic transition temperature toward a higher temperature could be attributed to the network structure in the LC matrix characterized by formed nematic domains that can induce local magnetic moments with a satisfactory number of neighboring LC molecules. The following interaction can then cause an increase in the transition point. The temperature behavior of LC composites near the transition temperature could be also explained by the fluctuation in order parameters and/or the increase in the bulk viscosity [26]. The reason for such behavior in any case should be related to H-bonds occurring in the SiO_2_ network.

It is evident that the results obtained using the SAW attenuation response measurement coincide substantially with the results of the light transmission measurements as well as the previous capacitance measurements. However, some dissimilarities could originate from the particularities of the used techniques. While the SAW attenuation measurement detects structural changes in some layers of the LC located just above the LiNbO_3_ piezoelectric substrate, the light transmission measurement responds to structural changes in any LC layer, including its center. In any event, both methods of investigation are able to individually contribute to the extension of knowledge about LC composite behavior, for which the connection of these results provides a more complex view of the studied problem.

## 4. Conclusions

In this contribution, light transmission measurements and the SAW technique were used to further study the memory effect in nematic liquid crystal 5CB composites with SiO_2_ nanoparticles and their effects by mixture with Fe_3_O_4_ nanoparticles. The study of 5CB composites confirmed the important role of SiO_2_ in their total structure, influencing the increase in the memory effect in comparison with pure LC, and on the contrary, repressing this effect by Fe_3_O_4_ nanoparticles. The registered memory effect was represented by hysteresis of both light transmission and SAW attenuation responses registered in the nematic phase. SiO_2_ nanoparticles, when mixed with LC, showed a marked improvement in the memory effect that increased with increasing nanoparticle concentration. The added Fe_3_O_4_ nanoparticles resulted in a decrease in the memory effect. Regarding the threshold voltage, it was found that this increased compared with pure LC in the case of both concentrations of SiO_2_ nanoparticles (1.0 wt.% and 0.5 wt.%); however, the addition of Fe_3_O_4_ nanoparticles shifted the threshold voltage in a different way. The anomalous shift in the threshold voltage in the case of different SiO_2_ concentrations can be influenced by the different structures of SiO_2_ domains and their environment created by both LC molecules and Fe_3_O_4_ nanoparticles. Measurements in the magnetic field also confirmed that while SiO_2_ aerosil markedly gives rise to the memory effect, the presence of Fe_3_O_4_ nanoparticles represses this effect, but in addition, it contributes to highlighting the 5CB magnetic properties, including oscillatory behavior. The other characteristic feature of the investigated composites was the increase in the transition temperature *T_NI_* caused by the addition of silica aerosil, upon which the additional presence of magnetic particles leads to a further transition temperature (*T_NI_*) increase. The presented results confirmed the potential of these composites for the application as non-volatile electro-optical memory devices suitable for data storage.

## Data Availability

Data are contained within the article.
